# On Turpeth Mineral in Croup

**Published:** 1846-05-01

**Authors:** Josiah Trowbridge


### On Turpeth Mineral, in Croup.

Communicated for the Buffalo Medical Association, by JOSIAH TROWBRIDGE, M. D.

In the January No. of the Buffalo Medical Journal, I noticed some remarks in relation to a communication from Doctor Hubbard, of Hallowell, Maine, on the use of Sub-Sulphate of Mercury, or Turpeth Mineral, in Croup. The Editor suggests, what I presume to be the fact, that the administration of this remedy in this disease is not confined to the practice of Doctor Hubbard, although I cannot find that it is recommended, or even alluded to, in our best standard works, as a remedy in Croup. On reading the notice, I was favorably impressed with the suggestion, from the effects of this medicine which I had noticed in my own practice. The administration of it, however, so far as I know, has been confined to desperate cases, where the ordinary remedies had failed to afford relief. In such cases it has sometimes failed, as might be expected, and in others it seemed to have the desired effect. I determined on the first opportunity to give it an early and fair trial; an opportunity occurred a few days afterwards.

*Case 1st.*—A fine healthy boy of four years old was attacked with croup about nine o'clock in the evening; some hive syrup with the ordinary domestic remedies was administered, with some apparent relief, but at about twelve o'clock, M. the day following, the symptoms had become greatly aggravated, at which time I was called in: I ordered tart. ant. grs. iv, ipecac grs. x, warm water ℥i; ʒii of the solution to be administered every fifteen minutes, until full emesis was produced. The emetic operated well. I saw the patient at five o'clock, and he seemed to be entirely relieved. The emetic not having operated as a cathartic, I directed proto-chloride hyd., grs. iv, ipecac gr. i, to be repeated in four hours, and to be followed with castor oil, if necessary. At twelve o'clock, P. M. I was again summoned to attend, and found my patient laboring under a renewed, and more violent attack than at noon. The cathartic had not operated. On my way, I had procured four leeches, but on my arrival I considered the patient to be in imminent danger of suffocation, from the rapid progress of the disease. I did not, therefore, deem it safe to wait the effect of leeching. I immediately administered grs. ii of the turpeth mineral, followed in fifteen minutes by gr. i, and at a further interval of fifteen minutes by an additional grain, in the whole, four grains. In from five to ten minutes from the administration of the first dose, the patient vomited violently, and threw up a large quantity of viscid mucus, exceedingly tough and adhesive. He vomited no more until the administration of the second dose, when, in addition to a quantity of mucus, he also threw up the contents of the stomach. He continued vomiting at intervals until four, A. M., at which time, he appeared to be in a great measure relieved. His bowels were moved at four, A. M., as I suppose by the action of the calomel given the evening previous. At eight o'clock in the morning, comfortable, slight cough and some hoarseness; ordered decoction of polygela senega.—Recovery rapid and complete.

*Case 2nd.*—I was called in great haste to see a child, four years old, at half-past nine in the evening, and was informed that the child was dying. On my arrival, I found the child in convulsions, and apparently asphyxiated. I learned that the child had exhibited symptoms of croup at nine o'clock the previous evening—had passed a restless night—that at five o'clock the previous afternoon, the attendants apprehended danger of the child's suffocating. As soon as the spasms attending the convulsion had so far subsided as to enable me to introduce a spoon into the child's mouth, I administered grs. ii of turpeth mineral; in fifteen minutes I gave another grain. In about five minutes from the administration of the first dose, the patient vomited violently, and threw off, as in the former case, a large quantity of tough viscid mucus; and at the second effort, the contents of the stomach, with more mucus. The patient was essentially relieved after the first act of vomiting, and of most of the urgent symptoms by twelve o'clock, P. M.; ordered proto chloride hyd. grs. iv, ipecac, gr. i to be repeated in four hours. At eight o'clock, A. M. cath. operated well—some cough, with slight hoarseness; ordered decoction polygela senega.—Recovery uninterrupted.

I used the turpeth mineral in two other cases with the like success. I concur with Dr. Hubbard in the opinion, that the medicine is a reliable, prompt, efficient, and safe emetic; and that it is free from the danger attendant upon the adminstration of large doses of antimony.

From the prompt manner in which it usually acts, I am inclined to the belief that its first effect is upon the mucus membrane of the fauces.

