# Erratum

**Published:** 1846-05-01

**Authors:** 


*Erratum.*—For "polygela Senega" page 271, read polygala Senega.

